# Alkaloids as Natural NRF2 Inhibitors: Chemoprevention and Cytotoxic Action in Cancer

**DOI:** 10.3390/ph16060850

**Published:** 2023-06-07

**Authors:** Darinka Gjorgieva Ackova, Viktorija Maksimova, Katarina Smilkov, Brigitta Buttari, Marzia Arese, Luciano Saso

**Affiliations:** 1 Department of Applied Pharmacy, Division of Pharmacy, Faculty of Medical Sciences, Goce Delcev University, Stip, Krste Misirkov Str., No. 10-A, P.O. Box 201, 2000 Stip, North Macedonia; viktorija.maksimova@ugd.edu.mk (V.M.); katarina.smilkov@ugd.edu.mk (K.S.); 2 Department of Cardiovascular and Endocrine-Metabolic Diseases and Aging, Italian National Institute of Health, Viale Regina Elena 299, 00161 Rome, Italy; brigitta.buttari@iss.it; 3 Department of Biochemical Sciences “A. Rossi Fanelli”, Sapienza University of Rome, Piazz. le A. Moro 5, 00185 Rome, Italy; marzia.arese@uniroma1.it; 4 Department of Physiology and Pharmacology “Vittorio Erspamer”, Sapienza University of Rome, Piazzale Aldo Moro 5, 00185 Rome, Italy; luciano.saso@uniroma1.it

**Keywords:** NRF2 inhibitors, alkaloids, plant bioactive compound, cancer, chemoprevention, anticancer therapy

## Abstract

Being a controller of cytoprotective actions, inflammation, and mitochondrial function through participating in the regulation of multiple genes in response to stress-inducing endogenous or exogenous stressors, the transcription factor Nuclear Factor Erythroid 2-Related Factor 2 (NRF2) is considered the main cellular defense mechanism to maintain redox balance at cellular and tissue level. While a transient activation of NRF2 protects normal cells under oxidative stress, the hyperactivation of NRF2 in cancer cells may help them to survive and to adapt under oxidative stress. This can be detrimental and related to cancer progression and chemotherapy resistance. Therefore, inhibition of NRF2 activity may be an effective approach for sensitizing cancer cells to anticancer therapy. In this review, we examine alkaloids as NRF2 inhibitors from natural origin, their effects on cancer therapy, and/or as sensitizers of cancer cells to anticancer chemotherapeutics, and their potential clinical applications. Alkaloids, as inhibitor of the NRF2/KEAP1 signaling pathway, can have direct (berberine, evodiamine, and diterpenic aconitine types of alkaloids) or indirect (trigonelline) therapeutic/preventive effects. The network linking alkaloid action with oxidative stress and NRF2 modulation may result in an increased NRF2 synthesis, nuclear translocation, as well in a downstream impact on the synthesis of endogenous antioxidants, effects strongly presumed to be the mechanism of action of alkaloids in inducing cancer cell death or promoting sensitivity of cancer cells to chemotherapeutic agents. In this regard, the identification of additional alkaloids targeting the NRF2 pathway is desirable and the information arising from clinical trials will reveal the potential of these compounds as a promising target for anticancer therapy.

## 1. Introduction

Cells are continuously exposed to exogenous stressors such as pollution, smoke, drugs, xenobiotics, ionizing radiation, and endogenous stressors derived by cellular metabolism. The presence of electrophiles or xenobiotics generates reactive oxygen species (ROS) which can induce alterations in the redox balance and promote cell death by damaging essential macromolecules such as lipids, proteins, and DNA. When ROS production increases or their scavenging by antioxidants decreases, cells undergo a process of oxidative stress [1,2]. If present in moderate concentrations, ROS are crucial for cellular life; indeed, they act as a second messengers in the transduction of extracellular signals and in the control of gene expression related to cellular proliferation, differentiation, and survival [3]. In fact, ROS have double importance in cancer. On the one hand, they can contribute to the occurrence of the modification of normal to cancer cells, on the other hand, a persistent oxidative stress renders cancer cells increasingly vulnerable to additional stressors and reverses drug resistance [4,5]. Elevated levels of ROS create conditions appropriate for the promotion of carcinogenesis through various mechanisms including generation of oxidative stress, which may favor oncogene activation, remodeling of tumor-suppressor genes, mitochondrial malfunction, and pro-inflammatory microenvironment [6,7]. In addition, cancer cells increase their unique antioxidative capacities [8,9,10,11]. In this way, cancer cells keep proliferation high and avoid ROS thresholds that would otherwise reduce the survival of cancer cells. In response to excessive ROS production, cancer cells activate several transcriptional programs for maintaining redox homeostasis [12,13,14,15,16,17]. 

Nuclear Factor Erythroid 2-Related Factor 2 (NRF2) is considered as the principal controller of cytoprotective activities in response to xenobiotics, electrophiles, and oxidants [5,18,19]. NRF2 plays important role in the control of oxidative stress, inflammation, mitochondrial function [20,21,22], and in the regulation of different genes. These genes are encoding antioxidant and Phase II metabolic enzymes and related proteins, linked to antioxidant response system (glutathione synthesis, xenobiotics detoxification, etc.) [23,24]. 

In healthy tissues, a transient activation of the NRF2 signaling pathway prevents cancer initiation by regulating several diverse transcriptional networks. More than 1000 genes possessing an Antioxidant Response Element (ARE) in their promoters can be activated by NRF2 during oxidative stress [25,26]. 

NRF2 is regulated by Kelch-like ECH-associated protein 1 (KEAP1), an adaptor subunit of Cullin 3-based E3 ubiquitin ligase (Cul3). Under normal conditions, KEAP1 binds to NRF2 in the cytoplasm and promotes the ubiquitination and proteasomal degradation of NRF2 and, thus, acts as a negative regulator of NRF2 [27,28,29]. In stress-inducing conditions, KEAP1, a cysteine-rich protein, readily reacts with intracellular ROS or electrophiles and alters NRF2/KEAP1 complex conformation thus inhibiting NRF2 ubiquitylation. Thus, when the interaction between NRF2 and KEAP1 is disrupted, proteasomal degradation of NRF2 decreases, causing de novo NRF2 synthesis within the cell and its increased translocation into the nucleus [30,31,32]. When NRF2 is imported to the nucleus, it heterodimerizes with small Maf proteins (sMaf) and binds to a regulatory enhancer sequence ARE, thus promoting the expression of antioxidant and detoxifying genes and down-modulating the production of pro-inflammatory mediators [21,33,34].

NRF2 is found in almost all cell types and tissues, but it is more abundant in tissues such as the intestine, lung, liver, and kidney, where detoxification reactions occur on site [35]. 

Recently, prooncogenic activity of NRF2 in cancer development, cancer progression, cancer cell growth and angiogenesis, and NRF2 involvement in anticancer therapy resistance is also under observation [2,36,37,38,39]. While NRF2 defends normal cells from oxidative stress, the excess of NRF2 in cancer cells may help them in adjusting to oxidative stress, surviving and resisting the redox environment (redox adaptation) [40,41]. In cancer cells, oncogenic signaling may affect the behavior of NRF2 by increasing its mRNA levels and generating constitutive NRF2 activation. The result is stress adaptation, drug resistance, cells proliferation, and activation of metabolic reprogramming and promotion of the expression of genes related to tumor progression. A cytoprotective role of NRF2 activation has been reported in many diseases associated with oxidative stress; thus, various natural sources (food and medicinal plants) have been tested for their ability to activate NRF2 and for their use as antioxidants and chemopreventive agents [42,43]. In many types of cancers, constitutive activation of NRF2 can be detrimental and responsible for cancer progression and chemotherapy resistance [44]; therefore, inhibition of NRF2 activity may be an effective approach for sensitizing cancer cells to anticancer drugs. Still, there is a scientific debate on whether activation or inhibition of NRF2 might represent an appropriate approach for the prevention and/or treatment of cancer. Regarding NRF2 modulatory effects, its activity as a detoxification agent of anticancer drugs (that possibly leads to chemoresistance) is well known; thus, favorable or adverse repercussions in cancer cells depend on a few factors, such as action control, tumor microenvironment, and cell type [19]. 

Among the known inhibitors of NRF2, alkaloids—natural bioactive components—are poorly investigated to date despite their pharmacological potential related to their high pro-oxidant and cytotoxic properties.

Alkaloids are secondary plant metabolites defined by Pelletier as a “cyclic organic compound containing nitrogen in a negative oxidation state which is of limited distribution among living organisms” [45].

Based on their complex structure and pharmacological properties, different classifications have been proposed. Several physiological pathways are involved in their biosynthesis. Most of them are derived from amino acids (tyrosine, tryptphan, ornithine, lysine, L-aspartate, anthranilic acid, and nicotinic acid), but there are also some classes of alkaloids that have been synthetized from steroids or terpenoids, and they are so-called pseudoalkaloids [46].

The most widely accepted method of classification of alkaloids is the chemical classification, where the main criterion is the presence of the basic heterocyclic nucleus (i.e., the chemical entity). According to their chemical structure, alkaloids have been grouped based on the nature of the heterocyclic ring in the following groups: mononuclear heterocyclic alkaloids represented as pyrrolidine alkaloids (hygrine) and aporphine alkaloids (boldine); polynuclear heterocyclic alkaloids given as tropane alkaloids (atropine); quinoline alkaloids (quinine); and Rauwolfia alkaloids (reserpine). Non-heterocyclic alkaloids have been found often in plants as atypical alkaloids and they are grouped in the phenylethylamine skeleton (ephedrine and capsaicin), tropolone skeleton (colchicine), and modified diterpene alkaloids (aconitine) [47].

The diversity in their chemical entity is the basic factor for the wide spectrum of associated pharmacological effects (antibacterial/antiviral activity, antitumor activity, antioxidant, anti-inflammatory, anticholinergic, neurological, and cardiovascular effects) [48].

In this review, we examine alkaloids from natural origin as inhibitors of the NRF2 pathway and as activators of tumor-suppressive properties to provide valuable information for the future development of alkaloid-based NRF2 inhibitors as a new therapeutic approach for the disruption of NRF2 oncogenic functions.

Systematic research of the literature was conducted by searching in the PubMed database for the keywords “NRF2 inhibitors” and “alkaloids”. The output is 148 retrieved results within the limit of 2000 to today; relevant articles were extracted, analyzed, and related linked articles were also considered.

## 2. The Cancer-Preventing Role of NRF2 

Numerous studies confirm the regulatory role of NRF2 as a reflection of its cytoprotective action in xenobiotic/oxidative stress. It is reported that this factor regulates the expression of several ARE genes, such as glutathione-S-transferase (GST), heme oxygenase-1 (HO-1), NAD(P)H: quinone oxidoreductase 1 (NQO1), and *γ*-glutamylcysteine ligase (*γ*-GCS), thus exerting a protective function [49,50]. For this reason, it is assumed that by inducing gene expression via NRF2, a detoxification from ROS occurs, which leads to mitigation of the cell oxidative damage. 

In addition, NRF2 was also shown to play a role in the response to inflammatory conditions. Namely, NRF2 was able to inhibit the oxidative-stress-derived activation of nuclear factor kappa-light-chain-enhancer of activated B cells (NF-kB) by decreasing ROS levels. NRF2 also inhibits its nuclear translocation, which in turn leads to a lowered expression of proinflammatory cytokines, as interleukin-1 (IL-1), interleukin-6 (IL-6), tumor necrosis factor alpha (TNF-α), and other mediators of inflammation [21]. Once its work has been completed, NRF2 is degraded in the cytoplasm, therefore allowing the cellular antioxidant defense to return to normal levels.

Indeed, the NRF2-related response to oxidative stress has been proposed in the pathogenesis of various diseases, such as various respiratory diseases [51], neurodegenerative diseases [52], inflammatory diseases [21], cardiovascular diseases [53], and metabolic diseases [54]. The NRF2 involvement in various types of cancer was also studied, including breast cancer [55], prostate cancer [56,57], urinary bladder cancer [58], hepatic carcinoma [59], pancreatic cancer [60], non-small cell lung cancer [61], and colorectal cancer [62]. 

The modulation of NRF2 can present an interesting option, both in the prevention and treatment of cancer as well as of other conditions, since its activation can lead to the promotion of antioxidant defense, a reduction in inflammation, detoxification of carcinogens, and inducing the apoptosis of cancer cells. 

## 3. The Cancer-Promoting Role of NRF2 and Its Role in Chemoresistance

**Figure 1:**
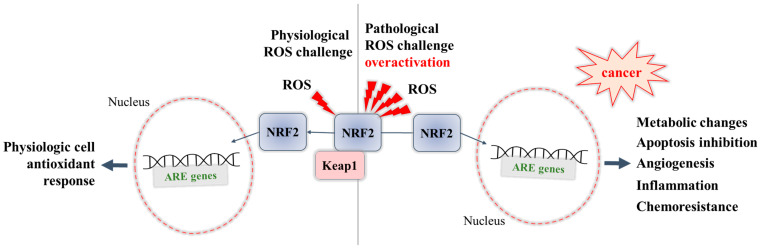
Dual role of NRF2: during physiological ROS challenge, and during pathological overactivation.

NRF2 is involved in increased sensitivity to a number of xenobiotic-induced toxicities and disease pathologies. An overactivation of NRF2 may lead to a negative impact, causing chemoresistance to therapy in cancer cells [63,64], thus highlighting the possibility of representing “a double-edged sword” [65]. The negative role of NRF2 highlighted by the promotion of the growth and survival of various types of cancer cells (NRF2-addicted/activated cancers) has been thoroughly studied and reviewed in the light of a deeper understanding of the mechanisms involved, and to explore the possibility of therapeutic approaches [66,67]. In these NRF2-addicted cell types, somatic mutation in NRF2 or its regulatory proteins (KEAP1) are on site, which blocks suppression of NRF2 levels (NRF2 is overexpressed and constitutively activated). These recurrent KEAP1 and NRF2 mutations (and in addition, missense mutations in Cul3) lead to metabolic reprogramming and allow cytoprotective action to cancer cells resulting in enormous proliferation and resistance to anticancer therapy [68]. Several mechanisms underlying the cancer-promoting role of NRF2 have been revealed (Figure 1):

- Overactivation of NRF2 in an ROS-enriched environment and participation in cancer cell proliferation and tumorigenesis. In various cancers, the cell metabolism increases in order to meet the demands of high cell proliferation, which in turn leads to the generation of ROS that aggravate oxidative stress and disturb the redox balance of cancer cells [57,69]. NRF2 is able to adapt itself to operate in a high-oxidizing environment, thus becoming overactivated. The accumulation of NRF2 in cancer cells was shown to promote their survival and proliferation and confer a support to tumorigenesis [69]. Some studies report that cancer cells can take control of the overactivated NRF2, with this factor even being a key player in the modulation of anabolic metabolism required for cancer cell growth and proliferation [70].
- Overexpression of NRF2 resulting in chemoresistance. Early studies have shown that overexpression of NRF2 is responsible for the resistance of cancer cells to chemotherapeutic agents as doxorubicin, cisplatin, and etoposide [71]. Recent studies have also reviewed the involvement of NRF2 in the activation of multidrug resistance proteins [72], in particular to modulate the expression of efflux transporters, of phase II metabolic enzymes [64], and of antiapoptotic genes (Bcl-2) [73,74], thus supporting the chemoresistance ability of cancer cells.

## 4. Inhibitors of NRF2—Potential Mechanisms

Hyperactivation of NRF2 in cancer cells can have significant implications for the tumor itself and its microenvironment. Being a part of important oncogenic and cytoprotective pathways in cancer cells, NRF2 modulates, directly or indirectly, carcinogenesis, proliferation, apoptosis, redox balance, formation of metastasis, and resistance to administered anticancer therapy. As a consequence of NRF2 elevation, the overexpression of drug-metabolizing enzymes, protein transporters, and redox-linked proteins is expected, which can lead to the undesired protection of cancer cells from the applied anticancer therapy [75]. For example, hyperactivation of NRF2 induces overexpression of genes of the multidrug resistance protein 1 (MDR1), breast cancer resistance protein (BCRP), and multidrug resistance-associated protein 1-5 (MRP1-5) and/or increased activity of chemoresistance-associated proteins [64,76]. Therefore, pharmacological targeting of NRF2/KEAP1 pathway with NRF2 inhibitors can be an attractive therapeutic approach for the disruption of NRF2 oncogenic functions. The exact mechanism of action of the NRF2 inhibitors is still unclear, and only a few mechanisms have been proposed in the corresponding studies (Table 1).

In contrast with the large number of compounds declared as NRF2 activators, very few compounds are recognized as NRF2 inhibitors; these ones have been mostly investigated as anticancer agents (direct or indirect, and in combinations), or as sensitizers of cancer cells to anticancer chemotherapeutics [33,67,102,103]. 

Among the known inhibitors of NRF2, alkaloids have recently aroused increasing interest as natural bioactive components, poorly investigated to date, but endowed with a high pharmacological potential for the contrast of cancer progression. 

There is a link between the effects (positive or negative) of alkaloids and ROS, causing the destruction of biomolecules or cell death. It is proposed that the base mechanism relies on the inhibition of NADPH-oxidase, the key enzyme directly involved in the reactions of intracellular ROS production. Besides the direct ROS production arising from the NADPH-oxidase catalytic activity, it is worth nothing that reactive species can also be obtained as an intermediate product of reactions catalyzed from other enzymes (e.g., xanthine oxidase). For some of the alkaloids, inhibition of the NADPH-oxidase relies on the NRF2 pathway activation. It is proposed that pro-oxidant behavior of alkaloids might be an important step for NRF2 activation. This depends on cell microenvironment factors, as some alkaloids can have dual role and act as antioxidants or pro-oxidants depending on intracellular conditions. Through NRF2, modest pro-oxidant activity of alkaloids can result in an observed antioxidant effect [104].

## 5. Plant Bioactive Compounds—Alkaloids as NRF2 Inhibitors 

Alkaloids as natural bioactive compounds have a significant role in developing anticancer therapy. Plant bioactive substances represent about 60% of the precursors of currently known anticancer agents. Well-known alkaloids such as vincristine and vinblastine have been presented as effective binders of tubulin and have been used in therapy of hematological and lymphatic neoplasms [105]. Additionally, colchicine in combination with taxane and *Vinca* alkaloids, or alone, displays elevated potentiality as a chemotherapy agent [106]. Berberine, an isoquinoline alkaloid, has shown anticancer properties, highlighted by the inhibition of cell proliferation obtained by interacting with respective microRNAs and suppressing telomerase activity [107,108]. 

On the other hand, piperine was shown to exhibit chemopreventive effects and anticancer activities [109]. Dhillon et al., in their study from 2014, investigated piperlongumine which is an alkaloid isolated from *Piper longum* [110]. They have shown that this alkaloid displays prooxidant effects and increases oxidative stress in the cells, while at the same time, it has only mild or negligible effect on antioxidant enzymes. It has been proposed, in this case, that oxidative stress might not be the crucial factor, as piperlongumine can directly interact with the nuclear exporter protein named chromosomal region maintenance 1 (CRM1). This exporter protein mediates the efflux of the nuclear factor, a forkhead box O transcription factor (FOXO1) from the nucleus. Transcriptome analysis performed on human pancreatic cancer cells treated with piperlongumine showed significantly enhanced mRNA expression of oxidative-stress-induced growth inhibitor 1, which is the protein directly linked to the NRF2 pathway [111]. Furthermore, one study investigated some synthetic analogues of piperlongumine as potential neuroprotective agents against hydrogen peroxide injury of the cells and it was found that the mechanism underlined was evidently NRF2-based [112].

### 5.1. Regulation of NRF2 and Cancer Metabolism

Relevant studies [70,113] showed the association between the NRF2/KEAP signaling pathway and the proliferation of cancer cells and tumorigenesis. The regulatory molecular mechanism of the NRF2/KEAP1 pathway against metabolic reprogramming in cancer has been recently reviewed [114,115], suggesting that regulation of the NRF2/KEAP1 axis might serve as a novel therapeutic strategy for cancer. 

We found many publications where alkaloids as naturally derived compounds were shown to be involved in the activation or inhibition of the NRF2 pathway, but only some of them took into consideration the regulation of this pathway in promoting cytotoxic or chemopreventive effects. The remodeling of cancer redox hemostasis or metabolic mechanisms may be provoked by few types of alkaloids [115]. 

A clear summary of data regarding alkaloids, their influence on NRF2/KEAP1 axis regulation, and their direct or indirect therapeutic/preventive effects will be deepened in the following sections of this review.

### 5.2. Mechanisms Triggered by Alkaloids in the Regulation of NRF2 Signaling Pathway 

Alkaloids as NRF2 activators promote a cytoprotective effect in normal cells. Many studies have suggested this effect [114,116]. NRF2 confers anti-carcinogenic activity in normal cells by preventing their progression to tumor cells and cancer metastasis. A study by Wu et al., [117] indicated that oxymatrine, an alkaloid isolated from *Sophora flavescens*, increases NRF2 levels and the phosphorylation of protein kinase B (Akt) and endothelial nitric oxide synthase (eNOS). The authors concluded that the overall pharmacological effects of oxymatrine prevented homocysteine-induced endothelial injury by activating Akt-eNOS-NRF2 signaling pathway. Corynoline, an alkaloid isolated from *Corydalis bungeana*, has shown significant anti-inflammatory effect by activating NRF2 signaling [118]. Another alkaloid compound, piperine, failed to modify ROS basal levels in unstimulated cells, leaving unchanged the transcription factor peroxisome proliferator-activated receptor delta (PPARδ), with antioxidant effects [119,120]. However, in another recent study conducted by Rehman et al. in 2020 [121], piperine exhibited inhibitory effects on the tumorigenesis of colon cancer cells by activation of the NRF2 pathway. In an ongoing clinical trial, the synergistic cancer-preventive effect of piperine and curcumine in early-stage prostate cancer patients is being investigated [122]. The main purpose of this trial is to determine whether the supplementation of piperine and curcumin can prevent or delay the progression of prostate cancer into a more aggressive cancer form.

Alkaloids as NRF2 inhibitors can act through two different ways:

1. A very few alkaloids known to date can exert direct cytotoxic effects on cancer cells. Many studies have reported that oxidative stress and redox homeostasis imbalance have been crucial factors in regulating tumor progression. While a small increase in ROS promotes cancer cell growth and survival, high levels of ROS could lead to activation of different cell death pathways and inhibit the tumorigenesis [123]. Therefore, inhibition of the NRF2 pathway leads to impairment of the NRF2-induced activation of antioxidant enzymes and promotes alternative cell strategies aimed at neutralizing high free radicals and may trigger different apoptotic pathways [1]. In this way, alkaloids (such as berberine, evodiamine, and diterpenic aconitine types of alkaloids, reported here later) can exhibit inhibitory effects on the NRF2 pathway and therefore exert cytotoxic effects in numerous tumor cell types.
2. Some recent studies have shown that alkaloids with NRF2 inhibitory activity can modulate cancer sensitivity for given chemotherapeutic agents and indirectly participate in cancer therapy [115,124,125]. Sustained activation of NRF2 can provoke resistance in chemotherapy on some tumor types, and in this view the degradation of *β*-transducin repeat-containing protein (β-TrCP)-NRF2 and KEAP1-NRF2 protein could be a potential methodology for enhancing chemosensitivity in cancer treatment [126]. The modulation of chemoresistance, a newly proposed mechanism, was investigated in a recent study where caffeine was examined as a purine alkaloid that decreases NRF2 levels (without affecting hypoxia-inducible factor-1α levels) and the expression of NRF2-targeted genes. Authors suggested that caffeine enhances doxorubicin-induced toxicity in A549 spheroids mediated by the reduction in Claudin-2 (a component of tight junctions) and NRF2 expression [127].

### 5.3. Alkaloids as NRF2 Inhibitors in Cancer Metabolism

According to study by Tan et al. from 2011 [128], berberine exhibited dual effects in cell survival since, while it is able to promote cancer cell death, it also protects normal cells from death. Many other studies have confirmed that berberine possesses anticancer effects. Guan et al., in an investigation from 2020 [129], examined the cytotoxic effect of berberine combined with evodiamine (an indole alkaloid) and their possible synergistic effects in colorectal cancer cells. Their results indicated that berberine inhibited the cell viability of CaCo-2 and HT-29 cells in a dose-dependent manner. Furthermore, evodiamine enhanced the cytotoxic effect of berberine, but only in CaCo-2 cells. The synergistic effects of evodiamine and berberine were further examined by a special software based on the Chou–Talallay method, which released a combination index < 1 (of 0.31–0.59), thus confirming the synergistic cytotoxic effect of the two alkaloids on CaCo-2 cell lines. However, the same synergistic effect was not observed in other colon cancer cell lines; precisely, this was the case of HT-29 cell line that lacks P-glycoprotein (P-gp). This finding leads to a conclusion that P-gp expression was responsible for the synergistic cytotoxic effects of berberine and evodiamine. They have shown that evodiamine attenuates the overexpression of the P-gp gene in a dose-dependent manner in berberine-treated cells. NRF2/P-gp axis association with clinicopathological characteristics in colorectal cancer was reported by Sadeghi et al. in 2018 [130]. Among the other functions, NRF2 is one of the key transcriptional factors for P-gp. They suggested that the inhibition of NRF2/ABCB1 signaling (where ABCB1 is a regulatory gene for P-gp) can be considered as a novel strategy to improve the efficacy of chemotherapeutics against colorectal cancer. Additionally, the study of Guan et al. [129], noted that cytoprotective effects of berberine on H9c2 cardiomyocytes from injury provoked by evodiamine were significant, and the mechanism underlying this effect was NRF2 accumulation in nuclear fractions. NRF2 accumulation was induced by berberine and decreased by evodiamine. 

Berberine, (in the form of berberine hydrochloride) was the subject of interest in a clinical trial [131] that investigated its chemopreventive effects with respect to the onset of colorectal cancer in patients previously identified as a group at high risk for developing adenomas. In particular, the experimental design foresaw a daily berberine hydrochloride intake of 300 mg. Once available, the results of this trial study, completed in 2021, will provide interesting information on the protective role of berberine. Another clinical trial [132] also investigated the preventive colorectal cancer role of berberine chloride in co-treatment with mesalamine in Chinese patients with ulcerative colitis. Berberine was shown to significantly decrease colonic tissue inflammation (measured by the Geboes score) [133]. Thus, berberine is receiving reasonable interest due to its anticancer activity based on many biochemical mechanisms, especially its proapoptotic and anti-inflammatory effect. Its capacity for sensitization and elimination of drug resistance are also very promising trends in the ongoing research. This is highlighted with the fact that berberine exhibits low toxicity towards healthy cells, which makes it safe for clinical use [134]. Indeed, there are data about berberine toxicity (developmental toxicity, immunotoxicity, cardiotoxicity), generally from animal models (rats, mice), which indicate that its toxicity depends on the dose, route, and duration of administration [135]. This is supported by the finding that the safety dosage of berberine in oral administration in mice is 20.8 g/kg which in humans would be extrapolated as 2.97 g/kg, which is 100 times above the usually prescribed doses in clinical trials studies [136].

Terpenoid molecules have been proposed as effective NRF2 inhibitors and potential chemotherapeutics. This was recently confirmed by Xiang et al. for brusatol [137], a triterpenoid lactone, which proved to be an effective inhibitor of cell invasion and migration via NRF2 pathway. For another bioactive diterpenoid, oridonine, the inhibitory effect of its terpenoid moiety on the NRF2 pathway was confirmed in human osteosarcoma cells, associated with the promotion of apoptotic effect, in vitro and in vivo [138]. 

These studies give a hint for investigating terpenoid alkaloids, where diterpenoid alkaloids have shown special ability with respect to NRF2 suppression. Li et al., in 2021, performed a detailed analysis of diterpenoid alkaloids isolated from *Aconitum sinomontanum*, and besides the already known compounds, they found some new C-19 forms of diterpenoid alkaloids with inhibitory potential on NRF2 [139]. The highest inhibitory effect on the expression of NRF2-regulated genes was exhibited by the compound labeled as number 16 in this study, previously confirmed as delvestidine [140], with an inhibition rate of 25% at the concentration of 20 µM.

Other compounds with slight effects on the expression of NRF2-regulated gene were also examined. The structure labeled as number 2 in this study, a rare rearranged aconitine-type C-19 diterpenoid alkaloid, showed a slight inhibitory effect (10% inhibition rate) on the expression of NRF2-regulated genes at concentration of 10 µM and therefore promoted cytotoxic activity [139]. Apart from the promising NRF2-inhibitory activity, the present research in this field is limited concerning risk factors, particularly regarding the possible cardiac toxicity [141,142]. Thus, future studies should focus on increasing the selectivity of the cytotoxic action on cancer cells while sparing the healthy cells.

In a recent study from 2021, Fouzder et al. [125] reported that trigonelline, an alkaloid derived from niacin metabolism [143], used as a pretreatment of NSCLC cells (A549 and NCIH460) at 50µM for 1 h caused a decline in the nuclear pNRF2-level after 12 h. Additionally, using immunoblot analysis, they observed that trigonelline (50 μM) treatment for 24 h did not cause any change in the total cellular NRF2 protein level. However, the protein levels of NRF2 target genes NAD(P)H dehydrogenase quinone 1 (NQO1) and heme oxygenase 1 (HO1) clearly declined after trigonelline treatment, thus indicating that the non-availability of nuclear NRF2 was the cause for their decreased expression. Furthermore, they showed that trigonelline inhibits NRF2 activation and cell proliferation via blocking the activation of the epidermal growth factor receptor (EGFR) signaling pathway. The most important conclusion of the study regarded the observed combinatorial effect of trigonelline-cisplatin and trigonelline-etoposide that resulted as a dose dependent synergistic cytotoxicity in lung cancer cells. Similar results were obtained by Tazehkand et al. [124], which confirmed the NRF2 inhibitory effects in overcrossing the oxaliplatin resistance in colon cancer cells. To examine the contribution of trigonelline in the suppression of the chemoresistance, they have used trigonelline-loaded micelles and they have shown that trigonelline in different concentration promoted NRF2 inhibition, followed by enhanced oxaliplatin-induced cytotoxicity. Toxicological studies of trigonelline were performed mainly using animal models and cell cultures, and only few human data exist. Acute human toxicity was predicted in silico using the computational platform ProTox-II (with a prediction accuracy of 69.26%) where the results were negative. Furthermore, no reports for subchronic toxicity in humans have been published [144]. Concerning its safety data, trigonelline is an important candidate as a potential anticancer agent in future studies.

Although a small number of NRF2 inhibitors have been identified so far, in comparison to the large number of natural NRF2 activators, these compounds could have different effects in cell survival. Overexpression of NRF2 provides a growth advantage for cancer cells and generally protects those cells from oxidative stress and anticancer agents, thus contributing to chemoresistance. Therefore, some natural-origin alkaloids acting as NRF2 inhibitors promote augmentative cell sensitivity of cancer cells against chemotherapeutic agents.

## 6. Conclusions

Alkaloids have always attracted scientific interest due to their potential positive, but also toxic effects on human health. The precise mechanism of the action of alkaloids in inducing cancer cell death or promoting the sensitivity of cancer cells to chemotherapeutic agents is either unknown or not specifically determined. The connection that links alkaloids with oxidative stress pathways and NRF2 modulation is strongly supported. Increased nuclear translocation of transcription factor NRF2 and an increase in the de novo synthesis of NRF2 may represent one of the main mechanisms exerted by alkaloids in cancer cells. The recruitment of other nuclear factors with antioxidant effects, such as FOXOs and PPARs by alkaloids, can synergize with the NRF2 pathway and have an impact on the synthesis of endogenous cell antioxidants and antioxidant enzymes. Another possible mechanism related to alkaloids as potential anticancer agents may be ascribed to their pro-oxidative effects. 

Therefore, future work should be directed towards taking into account the overall effects of alkaloids on cancer cells, considering both their positive (there is an inhibition) and neutral (there is no inhibition) effects. The first accepted mechanism as a linkage between NRF2 and anticancer potential was the induction of antioxidant enzymes. Through this mechanism, NRF2 exerts a protective role in healthy cells against xenobiotics, including carcinogens, by overexpression of cytoprotective genes. At the same time, in cancer patients, this mechanism can be related to deleterious effects, manifesting cancer progression and chemoresistance. In many studies, NRF2 holds a central place as being responsible for anticancer drug resistance and the reduced drug-sensitivity of cancer cells in patients undergoing chemotherapy. Since many types of cancers depend on NRF2-mediated cytoprotective actions to oppose and compensate for the negative conditions in their microenvironment, the pharmacological targeting of NRF2 is expected to potentiate and increase the efficacy of anticancer therapy. 

Based on these findings, the use of alkaloids as NRF2 inhibitors might be a promising approach in anticancer therapy. In this regard, additional alkaloids targeting NRF2 pathway should be identified and tested in clinical trials.

## Figures and Tables

**Table 1:** Proposed key mechanisms of action of the known NRF2 inhibitors.

No.	Proposed Mechanism	Compound (NRF2 Inhibitor)	References
1.	Stimulation of NRF2 poly-ubiquitination and NRF2 degradation (proteolysis of NRF2, downregulation of NRF2), induction of apoptosis	Brusatol (triterpenoid lactone), berberine (alkaloid), convallatoxin (cardenolide glycoside)	[77,78,79,80,81,82,83,84,85,86]
2.	NRF2 mRNA degradation, reduction of NRF2 binding to AREs, downregulation of NRF2-target genes	Luteolin (flavonoid), parthenolide (sesquiterpene), cordycepin (adenosine derivative)	[87,88,89,90,91,92,93]
3.	Prevention of nuclear translocation of NRF2, decrease in the mRNA and protein levels of NRF2, inhibition of NRF2/ARE pathway	Trigonelline (alkaloid), retinoic acid (metabolite of vitamin A), chrysin (flavonoid), wogonin (flavonoid)	[94,95,96,97,98,99,100,101]

## Data Availability

No new data were created or analyzed in this study. Data sharing is not applicable to this article.
